# Something More and Important about Dr. Taggart and the Dental Profession

**Published:** 1909-04-15

**Authors:** Edmund Noyes

**Affiliations:** Chicago


### SOMETHING MORE AND IMPORTANT ABOUT DR. TAGGART AND THE DENTAL PROFESSION.

BY EDMUND NOYES, D.D.S., CHICAGO.

To the older men of Illinois, who for thirty years have been witnesses of Dr. Taggart's readiness to make his professional brethren acquainted with everything he knew and everything he could do, the present antagonism between him and the profession seems very deplorable, and every possible effort should be made to bring about agreement and co-operation instead of antagonism. There has been considerable complaint and criticism of Dr. Taggart, much of it by men who have not bought his machines and have no claims upon him; we are at present more concerned with the attitude and the duty of the profession toward him. There is essential and important misunderstanding by the profession generally as to Dr. Taggart's attitude, intentions and efforts as expressed in the suit he has brought for the protection of his patents. This will be best shown by a brief account of the facts, the principles and the duties relating to the subject. First, as to patents; the dental code of ethics has nothing to say about patents either directly or by implication. "The Principles of Medical Ethics" is explicit and sweeping, in a short clause as follows: "It is equally derogatory to professional character for physicians to hold patents for any surgical instruments or medicines." The attitude of the dental profession is that of tolerance, probably approval, of the patenting of such things as can be made and sold in the open market by the makers or the supply houses. This is evident from the fact that many men holding such patents have never had their membership or standing in dental societies called in question. The profession has shown, and rightly, as I believe, an uncompromising dislike and opposition to such patents as can only be enforced or protected by the collection of an annual office license or of royalties on the operations performed. These are sometimes called "process patents." This opposition is little, if at all, on account of any unwillingness that one who gives to the profession some valuable new process or operation should be suitably rewarded financially. It is chiefly on three grounds. First, because the exactions are likely to be extortionate, as was the case by the Dental Vulcanite Co., and attempted by the Crown and Bridge Co.; second, because the manner of collection is vexatious and irritating; third, and perhaps most justly, because the larger part of the money collected is likely to go to people outside the profession, who have conferred no benefit upon us and to whom we are under no moral obligation. In the case of the Vulcanite Co. this happened as to the whole amount collected.

Dr. Taggart's attitude and intentions in these matters ought to be inferred and understood by all the older men of Illinois by the illustrations of it they have seen in him during the past thirty years. It is, however, shown more positively by an occurrance that happened in July 1906, an account of which has not heretofore been published. At that time, long before his patents were granted and half a year before the public announcement of his process, Dr. Taggart received a letter, (which I have read), in which a perfectly responsible business man of Chicago proposed, with some of his friends, to form a corporation and take over Dr. Taggart's patents when they should be granted, with such improvements as he might make subsequently, and to pay Dr. Taggart $100,000.00 cash and one-fourth of the stock. He refused the offer because he was unwilling to put it in the power of men outside the profession whom he could not control, to exploit the profession after the manner of the Vulcanite Co.

At the present moment the dental profession are exploiting Dr. Taggart to such an extent that he is the only man in the profession who has not profited financially by the use of his process. It is perhaps natural that the men who are doing this should have some wholesome fear that he may retaliate if he subsequently should have the power to do so, but in view of his character and record he should not be accused of it until he begins to do it. When Dr. Taggart took his patents he was advised that he could not defend the patent on his machine without also taking a patent on the process. What Dr. Taggart wishes to do is to sell his machine and to receive by that means his reward for what he has given to the profession. He has never asked anyone for a license fee or royalties for the use of his process, and his present suit is not for that purpose but only to prevent the defendant from using his process except with his machine. Now, the purchase of the machine carries with it, not as a favor or by agreement but by necessary legal implication, all the rights and privileges under both patents for their entire term. It has been called a mistake for Dr. Taggart to make the price of his machine so high. That may or may not be true as relates to his business interests. As it relates to the profession, if there is any man who thinks he would be casting inlays except for Dr. Taggart, let him speak up and tell us from what other source he feels sure that he would have derived the practice, and if there is any man who thinks it will not be worth $110.00 to him and his patients to cast inlays, crowns and bridges during the next fifteen or sixteen years let him speak out. He would probably be laughed out of court, or if not, perhaps those who think it is worth more would take up a collection to supply the deficiency.

There appears to be a general demand on the part of the profession that Dr. Taggart withdraw his suit and rely upon the generosity of the profession to compensate him for his sacrifices and expenses in giving the casting process to them. If during the eight months from the time his machines were ready and before the bringing of his suit there had been any adequate disposition to do justice to Dr. Taggart, leaving out generosity, no suit would have been brought. If a man owed you a debt which he acknowledged but refused or neglected to pay and you brought suit believing you could collect it would you withdraw the suit and trust his generosity to pay the debt afterward? That appears to be exactly what the profession is asking of Dr. Taggart.

The present attitude of the profession puts Dr. Taggart "between the Devil and the deep sea." If he loses his suit to maintain his patents they propose, (judging by the experience of what the profession has done in this case and in other cases in the past) to make him a martyr financially, and if he wins his suit they intend to make him a martyr professionally for holding a process patent. The solution of the situation is simple and the moves for it are due from the members of the profession individually. If we do not like the process patent, let us sustain the patent on the machine by buying it so largely that the process patent can be left in disuse.

It is a maxim of law which applies as properly before the bar of professional judgment and opinion as before a United States Court, that, "He who comes into court asking for justice must himself do justice", in other words "H e must come with clean hands." How can the members of the profession come before the bar of professional judgment asking Dr. Taggart to relinquish his legal rights and depend solely on the generosity of his profession while denying him the justice they all admit to be due him? Or how can any man plead for the maintenance of ethical standards unless he deals uprightly himself? The adopted code does not include all of ethics, it was not thought necessary to say "Thou shalt not refuse to pay just debts."

It has been suggested and some men have seemed inclined to promise that if Dr. Taggart will withdraw his suit the profession will rally generously to his support. Possibly this is true, but, in view of their not having done so before he was forced to commence suit to establish his right, there seems reasonable doubt that they would do so now. However, there should be shown on the part of the dental profession an honest effort, even at this late date, to treat the matter fairly and deal justly, knowing Dr. Taggart as I do, I believe he would gladly meet the profession if necessary even more than half way in an honest effort to remove all difficulties between him and the profession, or that part of the profession which seems to think Dr. Taggart is wrong in the position he has taken.

The remark has been made to me "We do not like the idea of being forced to buy Dr. Taggart's machine." It is a common sentiment among honest men that they do not like to be forced to pay their debts; they therefore pay them voluntarily. We owe Dr. Taggart a great sentimental debt, which is to be paid in gratitude, affection, honors and fame, but we owe him just as truly a debt in money for money value received and if we refuse or neglect to pay it we ought not to be any more surprised if we find ourselves defendants in suits at law than we would be if our butcher or grocer were to sue us for a debt we refused to pay.

